# Ibrutinib-Induced Cardiac Tamponade in Chronic Lymphocytic Leukemia

**DOI:** 10.4274/tjh.galenos.2020.2020.0446

**Published:** 2021-02-25

**Authors:** Keisuke Kidoguchi, Yasushi Kubota, Yuki Nishimura, Haruna Kizuka-Sano, Shinya Kimura

**Affiliations:** 1 Saga University, Department of Internal Medicine, Saga, Japan

**Keywords:** Ibrutinib, Cardiac tamponade, Bleeding, Atrial fibrillation, CLL

## To the Editor,

Acute cardiac tamponade is a rare but fatal disease that requires urgent intervention. The underlying cause of acute cardiac tamponade varies widely [1]. If the pericardial effusion is bloody, the cause is most likely iatrogenic (31%), a complication of invasive cardiac procedures [2]. Another major common cause is malignancy (26%). As a paraneoplastic complication, half of the cases are related to lung cancer; hematologic malignancies are rare (4.8%) [3]. Both bleeding and atrial fibrillation (AF) are well-known adverse effects of ibrutinib [a Bruton’s tyrosine kinase (BTK) inhibitor], but acute cardiac tamponade without concurrent use of antiplatelet or anticoagulant therapy has not been recognized [4,5]. This is a case report of cardiac tamponade induced by ibrutinib without concurrent use of antiplatelet or anticoagulant therapy.

**Figure 1:**
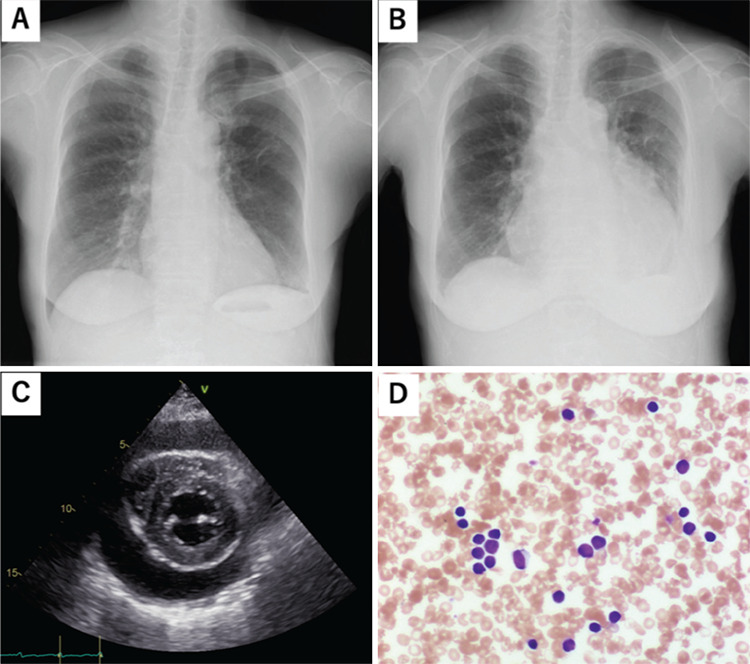
**A, B)** Chest X-ray revealed marked cardiomegaly (B), which was not apparent 2 months prior (A); **C)** transthoracic echocardiography showed a large pericardial effusion with right ventricular collapse; **D)** the drained bloody pericardial effusion contained many CLL cells. CLL: Chronic lymphocytic leukemia.

A 70-year-old woman with a 12-year history of chronic lymphocytic leukemia (CLL) visited our hospital complaining of malaise and dyspnea. She had not been prescribed antiplatelet or anticoagulant agents. No bleeding diathesis or cardiac disease had been identified. Her initial treatment for CLL was cyclophosphamide monotherapy, which was discontinued because of progression of the disease. The treatment was changed to ibrutinib monotherapy and she achieved partial remission. On admission, she was afebrile, but her respiratory rate was increased to 24 breaths/min and her systolic blood pressure was 96 mmHg. On physical examination, jugular vein distention was prominent and heart sounds were muffled. Laboratory tests showed a leukocyte count of 43x10^9^/L with 89% abnormal lymphocytes, a hemoglobin level of 102 g/L, and a platelet count of 16.8x10^9^/L. Coagulation tests, including prothrombin time, international normalized ratio (1.09), and partial thromboplastin time (30.4 s), were normal. An electrocardiogram showed AF with low QRS voltage. A chest X-ray showed marked cardiomegaly (Figure 1), which was not apparent 2 months earlier. Transthoracic echocardiography showed a large pericardial effusion with right ventricular collapse, consistent with cardiac tamponade (Figure 1). Emergent pericardiocentesis was performed and 355 mL of bloody fluid was drained. Fluid analysis findings were as follows: protein, 4.1 g/dL; lactate dehydrogenase, 2100 U/L; and leukocytes, 4.2x10^9^/L, with 42% lymphocytes. The lymphocytes were small, with clumped chromatin and scanty cytoplasm (Figure 1). Flow cytometry analysis showed that the lymphocytes were positive for CD5, CD23, and CD19, which was consistent with CLL. Ibrutinib was discontinued and the symptoms resolved without recurrence of AF or the pericardial effusion.

In this case, cardiac tamponade and AF were ibrutinib-associated adverse events because they improved immediately after ibrutinib discontinuation. The mechanism of bleeding is due to the off-target effect of ibrutinib hampering platelet aggregation [6]. Ibrutinib inhibits platelet aggregation in a collagen-mediated manner and is solely dependent on the physiological half-life of platelets (3-4 days) [7]. Ibrutinib irreversibly inhibits BTK, but it has a short half-life (<3 h). As a consequence, bleeding is considered to be a reversible adverse effect. Although AF and bleeding events are common adverse events associated with ibrutinib, cardiac tamponade is rare. If patients on ibrutinib complain of symptoms such as dyspnea and palpitations, not only AF but also cardiac tamponade should be considered.
